# Editorial: User-Friendly Tools Applied to Genetics or Systems Biology

**DOI:** 10.3389/fgene.2020.00985

**Published:** 2020-09-09

**Authors:** Helder I. Nakaya, Juilee Thakar, Vinicius Maracaja-Coutinho

**Affiliations:** ^1^Department of Clinical and Toxicological Analyses, School of Pharmaceutical Sciences, University of São Paulo, São Paulo, Brazil; ^2^Department of Microbiology & Immunology, University of Rochester, Rochester, NY, United States; ^3^Department of Briostatistics & Computational Biology, University of Rochester, Rochester, NY, United States; ^4^Advanced Center for Chronic Diseases – ACCDiS, Facultad de Ciencias Químicas y Farmacéuticas, Universidad de Chile, Santiago, Chile

**Keywords:** user-friendly tools, systems biology, omics analyses, bioinformatics and computational biology, computational tool and servers

Life scientists now have access to an unprecedented amount of experimental data. A single laboratory can measure the levels of all transcripts, proteins, or metabolites of an organism under different perturbations or can sequence the entire genome of hundreds of individuals or specimens. Systems biology aims to study the behavior and interaction of these molecules, using advanced mathematical models. Modern data-intensive genetics is also often dependent on statistical tools for identifying signals through population-level measurements. However, according to Sydney Brenner “we are drowning in a sea of data and starving for knowledge. Today, biology is more about gathering data than hunting down new ideas.” This is partly due to the fact that a substantial number of researchers who are capable of thinking about new insights, are not able to deal with the vast amounts of data generated by modern technologies. This Research Topic aimed to help those researchers interested in analyzing high-throughput data, but lacking knowledge on programming languages or bioinformatics skills. With the collaboration of computer scientists and software developers, this issue brings an interesting collection of user-friendly tools with broad applications in genetics and systems biology.

As the first layer of biological information, the DNA carries the genetic instructions for the fine-tuning functioning of all known organisms. In this context, Sariya et al. performed a benchmarking of reference panels and tools for rare variants imputation in genome-wide association studies (GWAS) in admixed populations. Thus, Sariya et al. study will facilitate the selection of panels, tools, and parameters for rare variant imputation in GWAS. Related to prokaryotic genomes, two user-friendly tools were described with the purpose of performing comparative genomics analyses focused on Bacteria. Gene Tags Assessment by Comparative Genomics (GTACG) performs pan-genome comparative analyses (Santiago et al.) by identifying homologous genes and defining the gene families, followed by the documentation of the core/accessory genome, phylogenetic analysis and data visualization in an easy-to-use graphic interface. PhageWeb is web service for identifying prophage regions and for characterizing bacterial genomes (de Sousa et al.).

The analysis of the transcriptome, i.e., the set of all transcripts of a cell or tissue, provides an overview of the processes and signaling pathways related to diseases and various biological conditions. Four user-friendly computational tools described here (Pipeliner, ABioTrans, Simplicity DiffExpress, and MDP), facilitate the processing and analysis of RNA-seq data. By combining the Anaconda package manager with Nextflow scripting language, Pipeliner enable users to generate modular computational workflows for processing various types of sequencing data, including single-cell expression data (Federico et al.). ABioTrans is a web-browser based user interface that allows users to not only directly read RNA-Seq data files deposited in the GEO database, but also to perform dimensionality reduction, differential expression analysis, and gene ontology classifications (Zou et al.). After the raw RNA-seq data is summarized in read-count tables, Simplicity DiffExpress can be used to run differential expression analysis and to determine a bespoke statistical model validation for it (Palu et al.). Often, however, the huge heterogeneity among individuals can impact gene expression analyses. MDP webtool uses a dynamic interface to inspect gene expression data and identify samples that are potential biological outliers (Gonçalves et al.). It is also useful to identify subgroups of patients classified with a particular disease but with different expression profiles or to reveal particularities of distinct illness that are perturbing the expression of genes or pathways.

The integration of the set of transcripts, proteins or metabolites in a particular condition using network approaches allows analysis beyond just one genes/protein. The webCEMiTool provides an easy-to-use environment for identifying gene co-expression modules, followed by their functional characterization through the automatic integration of gene-to-gene or protein-protein interaction networks, gene set enrichment analysis and overrepresentation of pathways or ontologies (Cardozo et al.). The FindTargetsWEB focused on analyzing genome-scale metabolic networks of bacteria in order to identify potential therapeutic targets (Merigueti et al.). It searches for fragile genes available in the network, in which its blockage could impair one or more metabolic functions.

BioNetStat provides a user-friendly environment for the comparison of two or more networks simultaneously, by exploring different topological features available in each network (Jardim et al.). The review from Ramos et al. provides a very interesting and intuitive explanation of the key concepts and terminology behind network biology, as well as a didactic guide on how to perform network analysis using user-friendly tools.

After integration, users can develop or simulate biological models representing the living system of the studied organism in particular conditions of interest. In this context, Afshar et al. generated a model in CellML format for glucose uptake in the epithelial cell of the small intestine (enterocyte). This model structure permits different changes in the components and parameters, facilitating its reuse and customization. Ii et al. developed a tool, named XitoSBML, that helps the users to automatically generate Systems Biology Markup Language (SBML) Level 3 Version 1 spatial model files from microscopic cellular images (Ii et al.). The converted model holds molecular concentrations, locations and biochemical reactions, which can be used by SBML-supported simulators to perform spatial simulations based on the generated model.

Finally, after walking from genomes to systems/networks and the computational modeling of living systems, the user might be interested in store or explore its information in biological databases. In this Research Topic two databases for genetics and systems biology data organization were described. croFGD integrated genomic information and dozens of RNA sequencing data from different tissues and biological conditions of *Catharanthus roseus*, a medicinal plant with pharmacological activities, in order to build a functional genomics database (She et al.). It provides annotations, expression data, and network models (e.g., co-expression, protein-protein interactions, microRNA-target interactions), which can be explored dynamically through a web searchable interface and a set of tools for data analyses specifically for this species. Ma et al. developed a similar database for Moso bamboo (*Phyllostachys edulis*), the most economically valuable bamboo in Asia, called BambooNET.

A fundamental characteristic for a tool to be adopted widely by life scientists is that it should be user-friendly. Even if the application is specific to a small area of knowledge, the software needs to be easy to run by researchers without advanced knowledge in programming or statistical tools. As a trade-off, user-friendly versions generally have fewer parameters and adjustments than versions which are run on scripts or command lines. We hope that the reader will find a useful collection of such tools for genetic or systems biology research, democratizing bioinformatics and computational biology to a broad group of users with lesser computing background.

## Author Contributions

All authors listed have made a substantial, direct and intellectual contribution to the work, and approved it for publication.

## Conflict of Interest

The authors declare that the research was conducted in the absence of any commercial or financial relationships that could be construed as a potential conflict of interest.

